# FMT intervention decreases urine 5-HIAA levels: a randomized double-blind controlled study

**DOI:** 10.3389/fmed.2024.1411089

**Published:** 2024-10-18

**Authors:** Lihong Wang, Lianhu Yu, Zhiyue Liu, Chao Che, Yu Wang, Yongheng Zhao, Mengna Zhu, Guang Yang, Aihua Cao

**Affiliations:** ^1^Department of Pediatrics, Shandong University Qilu Hospital, Shandong, Jinan, China; ^2^Department of Pediatrics, Chinese PLA General Hospital, Beijing, China

**Keywords:** autism spectrum disorders, fecal microbiota transplantation, urine metabolomics, 5-hydroxyindoleacetic acid, 5-HT

## Abstract

**Background:**

Autism spectrum disorder (ASD) is often linked to gastrointestinal issues and altered serotonin metabolism. Emerging evidence suggests gut microbiota influence both, with fecal microbiota transplantation (FMT) offering a potential therapeutic approach. However, its impact on serotonin metabolism and ASD symptoms is not well understood. In this study, we aimed to evaluate the clinical effects of FMT and examine changes in specific urinary metabolites in children with ASD.

**Methods:**

A randomized double-blind controlled trial was performed to evaluate the clinical effects of FMT on GI and ASD-related symptoms. Gastrointestinal symptoms were assessed using the Gastrointestinal Symptom Rating Scale (GSRS), and the ASD-related symptoms were assessed using the Childhood Autism Rating Scale (CARS), Aberrant Behavior Checklist (ABC), and Social Responsiveness Scale (SRS) scores. Urinary metabolites were analyzed by homogeneous enzyme immunoassay using commercially available kits.

**Results:**

Significant improvements in GI and core ASD symptoms were observed following FMT intervention. The average GSRS scores decreased from 30.17 (before) to 19 (after; *p* < 0.0001), CARS scores decreased from 36.22 to 33.33 (*p* < 0.0001), SRS scores decreased from 151.17 to 137.5 (*p* = 0.0002), and the ABC scores decreased 76.39 to 53.17 (*p* < 0.0001) in the FMT group. However, in the placebo group, GSRS, CARS, and SRS scores showed no significant changes, while ABC scores decreased from 72 to 58.75 (*p* = 0.034). The FMT group also showed a significant reduction in urinary 5-hydroxyindoleacetic acid (5-HIAA) levels from 8.6 to 7.32 mg/L (*p* = 0.022), while other metabolites showed no significant changes.

**Conclusion:**

FMT is a safe and effective treatment for improving GI and core symptoms in children with ASD, with 5-HIAA showing potential as a urinary biomarker for treatment response.

## 1 Introduction

Autism spectrum disorders (ASDs) are neurodevelopmental disorders characterized by impaired social interactions, communication difficulties, and repetitive or restrictive behaviors that manifest in early childhood (1). The global prevalence of ASD has increased from 2.3 to 2.76% in recent years (2, 3). Although the exact pathogenesis of ASD remains unclear, both genetic and environmental factors, including gut microbiota dysbiosis, play significant roles (4, 5). Previous studies have demonstrated alterations in gut microbiota in children with ASD compared to normally developing children (6). Metabolic changes secondary to altered gut microbiota are considered important in understanding the pathogenesis of ASD (7). However, efficient biomarkers for the diagnosis of ASD have not been identified because of unclear underlying mechanisms. Current clinical management of ASD primarily relies on observational methods, standardized behavioral scales, and parental interviews (8). In addition, etiological treatments addressing the root causes of ASD are still under investigation. Given the prevalence of gut microbiota dysbiosis in ASD, fecal microbiota transplantation (FMT) has emerged as a potential therapeutic approach to restore gut microbiota homeostasis in patients with ASD. Several clinical studies have demonstrated the effectiveness of FMT (9, 10). Nevertheless, the precise mechanisms underlying its benefits remain uncertain (11).

Metabolomics studies in children with ASD have revealed various alterations in blood, urine, and fecal metabolites, including gut microbial co-metabolites, tryptophan and other amino acids, mitochondrial dysfunction-related molecules, and metabolites associated with the metabolism of purines and pyrimidines (12–15). For instance, a previous study using gas chromatography–mass spectrometry (GC/MS) identified different urinary metabolites, such as 3-(3-hydroxyphenyl)-3-hydroxy propionic acid (HPHPA), 3-hydroxyphenyl acetic acid (3-HPA), and 3-hydroxyhippuric acid (3-HHA), as potential biomarkers for gut microbiota dysbiosis in ASD. The study also showed significant alterations in the abundances of *Ruminococcus* and *Clostridium* species in children with ASD, suggesting that correcting these microbiota dysbiosis could reduce the levels of HPHPA, 3-HPA, and 3-HHA (16). In addition, studies have also shown that the metabolites, particularly, 3-HHA, metanephrine (MN), normetanephrine (NMN), HPHPA, 5-hydroxyindoleacetic acid (5-HIAA), homovanillic acid (HVA), vanillylmandelic acid (VMA), 3-hydroxyphenylpropionic acid (3-HPP), microalbumin (MALB), 17-hydroxycorticosteroids (17-OHCS), 17-ketosteroids (17-KS), and urine cortisol (UCOR), are altered after FMT treatments in Chinese children with ASD (17, 18). These studies indicate that monitoring specific metabolites could aid in diagnosing ASD. Recently, specific test kits for measuring altered urinary metabolites in children with ASD have been developed (12–18). In this study, we used the commercially available specific kits, which employ a homogeneous enzyme immunoassay (HEIA) method to assess the levels of 3-HHA, MN, NMN, HPHPA, 5-HIAA, HVA, VMA, 3-HPP, MALB, 17-OHCS, 17-KS, and UCOR.

We hypothesize that FMT will significantly improve ASD symptoms and result in measurable changes in urinary metabolite profiles. In this study, we aimed to assess the clinical efficacy of FMT against ASD in children. We employed a randomized double-blind controlled design to compare improvements in clinical scales and urinary metabolite changes between 41 children, with 19 receiving FMT treatment and the remaining children receiving a placebo. This study aims to provide insights into the metabolic effects of FMT and contribute to understanding its mechanisms of action.

## 2 Materials and methods

### 2.1 Patient recruitment

#### 2.1.1 Inclusion criteria and exclusion criteria

This study was approved by the Ethics Committee of Chinese PLA General Hospital. A total of 41 children (39 boys and 3 girls, aged 4–12 years) were recruited from Shandong University Qilu Hospital. All enrolled participants were diagnosed with ASD using the Autism Diagnostic Observation Schedule, Second Edition (ADOS-2). The participants were required to have not taken probiotics, antibiotics, or immunosuppressants for at least 1 month before enrollment in the study.

The exclusion criteria for the study included secondary autism caused by identifiable factors, such as metabolic abnormalities or genetic diseases, fever with a body temperature exceeding 37.3°C, serious gastrointestinal (GI) conditions that required immediate treatment (e.g., intestinal obstruction, bleeding, or perforation), severe immunodeficiency, a history of severe allergies, severe malnutrition or underweight, and children undergoing special dietary therapy.

Participants were randomly assigned to either the FMT or placebo groups using a random number table. This randomization process was carried out by a researcher who was not involved in recruitment or assessment, ensuring the integrity of the double-blinding process.

#### 2.1.2 Sample size calculation

The sample size calculation was based on the Childhood Autism Rating Scale (CARS) as the primary evaluative measure. The improvement threshold was set at a reduction of ≥2 points on the CARS scale. Assuming a sample standard deviation of 2, an alpha level of 0.05, and a beta level of 0.1 for a two-tailed test, the sample size was calculated using the paired *t*-test method. To account for a 40% attrition rate, the final sample size required was determined to be 19 participants per group.

### 2.2 Urine collection

Urine samples were collected in the morning into sterile containers that were not treated with preservatives. The exact time of collection was recorded. Parents transported the samples to the hospital on ice, and the samples were immediately cryopreserved at −80°C until analysis.

### 2.3 Fecal microbiota transplantation intervention

#### 2.3.1 Donor inclusion criteria

The donor for this study was an 11-year-old child with a BMI of 22.49 kg/m^2^. Blood cell analysis, liver and kidney function tests, electrolytes, and C-reactive protein levels were all within normal ranges. Pathogen tests were negative, and routine stool tests, including tests for *Clostridium difficile*, showed no abnormalities. The psychological status of the donor was assessed as good through various psychological scales and evaluations by professional physicians. No abnormalities were noted in the past medical or personal history of the donor.

#### 2.3.2 Fecal preparation

The FMT capsules used in this study were prepared by a professional microbiology laboratory, and the quality of the capsules was inspected. The FMT capsules were prepared through an anaerobic preparation process, including filtration and multiple centrifugation steps, from the fecal matter of the recruited donor. The processed fecal matter was then lyophilized and encapsulated. The placebo capsules, in contrast, were composed mainly of wheat bran and did not contain lyophilized powder of normal gut microbiota.

#### 2.3.3 Study design

All children with ASD participated in the study for a total of 9 weeks. This period included a 5-week phase of FMT or placebo treatment, followed by a 4-week follow-up observation. Before the FMT treatment, all participants with ASD were trained to swallow the capsules. In preparation for the FMT treatment, all participants with ASD followed a residue-free semi-liquid diet for 3 days to cleanse the bowels and reduce gastrointestinal motility, which promotes the colonization of the gut microbiota. In addition, they were administered GOLYTELY (polyethylene glycol) 1 day before the FMT treatment to ensure intestinal emptying. The FMT or placebo capsules were administered orally during the first week and again in the fifth week. Following the 5-week treatment phase, participants were monitored for an additional 4 weeks. Throughout the FMT treatment schedule, all participants continued with routine rehabilitation training.

### 2.4 Assessments of GI and autism symptoms

#### 2.4.1 Assessment of GI symptoms

To evaluate the improvement of GI symptoms, parents or guardians completed the GI symptom rating scale (GSRS). This scale includes 16 questions covering 5 domains: abdominal pain, reflux, indigestion, diarrhea, and constipation. Each question on the GSRS was revised to use a 7-point scale for more detailed responses. Assessments using the GSRS were conducted at baseline and the end of the experiment (at 9 weeks).

#### 2.4.2 Assessments and diagnosis of autism symptoms

In this study, we used ADOS-2, CARS, ABC, and SRS tools to assess and diagnose autism symptoms. ADOS-2 is a semi-structured interview tool used for the clinical diagnosis of autism and autism spectrum disorders. It evaluates five domains: communication, reciprocal social interaction, games/imagination, restrictive behaviors, and restricted interests. CARS is a 15-item scale designed to diagnose ASD and assess the severity of its symptoms. SRS is another assessment tool used to evaluate social responsiveness. It consists of 65 questions answered by parents or guardians, measuring aspects such as social awareness, social information processing capacity, reciprocal social communication, social anxiety/avoidance, and autistic preoccupations and traits. In addition, ABC was used to assess abnormal behaviors in children with ASD. It includes 58 items categorized into five areas: emotional lability/self-injury and aggression, social withdrawal/lethargy, stereotypy, hyperactivity, and inappropriate speech. The ADOS-2 was administered at baseline to diagnose ASD, while the CARS, ABC, and SRS were assessed at both baseline and 9 weeks.

### 2.5 Homogeneous enzyme immunoassay

#### 2.5.1 Reaction fundamentals

Homogeneous enzyme immunoassay (HEIA) involves a competitive reaction where small-molecule analytes in the sample compete with “small molecule–enzyme” conjugates for binding to specific antibodies. The binding of the small-molecule analyte to the antibody leads to the release of the conjugate, which catalyzes the conversion of NAD+ to NADH, resulting in a detectable change in absorbance at 340 nm. The concentration of the analyte is proportional to this change in NADH (19).

#### 2.5.2 Reagent components

Reagent 1 (R1) contained 55 mmol/L Trizma base, 0.112 mmol/L glucose 6-phosphate (G-6-P), 0.112 mmol/L *β*-nicotinamide adenine dinucleotide (NAD^+^), 0.85% sodium chloride, 3 mmol/L magnesium chloride, ≤0.2% rabbit polyclonal antibody against specific metabolites, 0.1% bovine serum albumin (BSA), and 0.05% preservatives (Preknin 300). The pH of the final solution was adjusted to 8.0.

Reagent 2 (R2) comprised 120 mmol/L Trizma base, 0.85% sodium chloride, 3 mmol/L magnesium chloride, ≤0.1% glucose hexaphosphate dehydrogenase–specific metabolites conjugate, 0.1% BSA, and 0.05% preservatives (Preknin 300). The pH of the final solution was adjusted to 8.2.

Calibration solution included 0.25% BSA, 0.85% sodium chloride, 50 mmol/L Trizma base, 0.05% preservatives (Preknin 300), 0.03% Tween-80, and 80.0–120.0 mg/L specific metabolites.

Quality control products comprised 0.25% BSA, 0.85% sodium chloride, 50 mmol/L Trizma base, 0.05% preservatives (Preknin 300), 0.03% Tween-80, 4.0–6.0 mg/L specific metabolites, such as 5-HIAA (level 1), and 40.0–60.0 mg/L specific metabolites (level 2). The pH of the final solution was adjusted to 7.0.

#### 2.5.3 Reaction process

The reaction process is conducted using a Beckman Company AU 680 fully automatic biochemical analyzer. Initially, 10 μL of the sample was introduced into the system. At time zero, 200 μL R1 was added, followed by 5-min incubation at 37°C. Subsequently, 50 μL R2 was added. The absorbance was measured at 340- and 405-nm wavelengths. The temperature was maintained at 37°C for 1.5 min, during which the absorbance reading, denoted as A1, was recorded. After an additional 3.5 min, a second absorbance reading, A2, was obtained, and the difference between A1 and A2 (ΔA = A2 − A1) was calculated. The reagent calibration involved six calibrators, each providing a ΔA value. The instrument used these values to generate a calibration curve by fitting the absorbance differences of the six calibrators. For sample analysis, the instrument calculated the concentrations of metabolites including 3-HHA, MN, NMN, HPHPA, 5-HIAA, HVA, VMA, 3-HPP, MALB, 17-OHCS, 17-KS, and UCOR based on the absorbance differences and the calibration curve.

### 2.6 Statistical analysis

The clinical and urinary metabolite data were analyzed using SPSS 25.0 software. Clinical scale data were described using mean values. To account for variations in urine volume that could affect metabolite concentrations, we calculated the concentration ratios of urinary metabolites to creatinine, rather than using the direct concentrations of urinary metabolites. The data meeting the normal distribution were analyzed using the *t-*test, while those deviating from the normal distribution were analyzed using the Wilcoxon test. Differences, with a *p*-value of less than 0.05 considered significant.

## 3 Results

### 3.1 Subject characteristics

A total of 41 children with ASD were enrolled in the study. All participants belonged to different families and were randomly allocated into two groups: One group received oral FMT capsules, while the other group received a placebo. All ASD participants completed the 9-week treatment and follow-up study. Due to challenges with compliance among children with ASD, urine samples could only be collected from 29 participants (15 from the FMT group and 14 from the placebo group). The characteristics of the participants at baseline are shown in Table 1. The groups were comparable in terms of age, gender distribution, body mass index (BMI), and mode of delivery. Furthermore, the CARS, Aberrant Behavior Checklist (ABC), and Social Responsiveness Scale (SRS) estimations showed no significant differences in autism-related symptoms between the two groups. The urinary metabolites are generally comparable at baseline levels (Table 2).

**Table 1:** Clinical characteristics of ASD participants.

Category	FMT group (*n* = 19)	Placebo group (*n* = 22)	*p*-value
Age	6.54 y	6.68 y	0.458
(Interquartile range)	(5.67, 7.33)	(5, 8.29)	
Gender			
Girls	1	2	0.639
Boys	18	20	
BMI	19.15 kg/m^2^	17.26 kg/m^2^	0.319
(Interquartile range)	(14.35, 21.9)	(14.43, 17.3)	
Mode of delivery			
Vaginal delivery	6	12	0.319
Cesarean section	13	10	
CARS Score	36.22	34.95	0.069
(Interquartile range)	(35, 38)	(32.5, 36)	
ABC	76.39	71.45	0.573
(Interquartile range)	(52.75, 105.75)	(59, 81.75)	
SRS	151.17	141.32	0.055
(Interquartile range)	(143.5, 164.5)	(130.75, 155.5)	

**Table 2:** Urinary metabolites between the two groups at baseline.

Clinical indicators	Placebo BSL	FMT BSL	*p*-value
	(*n* = 14)	(*n* = 15)	
3-HHA	19.12 (6.33, 16.72)	20.26 (6.72, 31.79)	0.59
MN	87.15 (71.16, 91.17)	116.72 (78.69, 107.95)	0.38
**NMN**	318.79 (238.69, 383.45)	229.28 (188.48, 269.04)	**0.03**
HPHPA	9.69 (4.69, 13.55)	18.25 (5.57, 25.14)	0.11
5-HIAA	11.42(7.1, 12.05)	8.6(7.27,9.58)	0.73
HVA	10.49 (8.4, 10.37)	9.02 (6.78, 10.79)	0.51
VMA	8.44 (6.36, 9.6)	7.64 (5.71, 10.01)	0.36
3-HPP	34.71 (11.95, 46.02)	48.06 (17.75, 73.24)	0.24
MALB	7.49 (3.29, 9.69)	8.67 (6.6, 10.62)	0.36
**17-OHCS**	3.35 (2.43, 4)	4.97 (3.38, 6.59)	**0.02**
17-KS	0.25 (0.005, 0.3)	0.37 (0, 0.5)	0.96
UCOR	154.03, (104.24, 193.07)	221.95 (153.5, 267.7)	0.12

NMN: Normetanephrine, 17-OHCS: 17-Hydroxycorticosteroids.

### 3.2 GI and ASD evaluations

**Figure 1:**
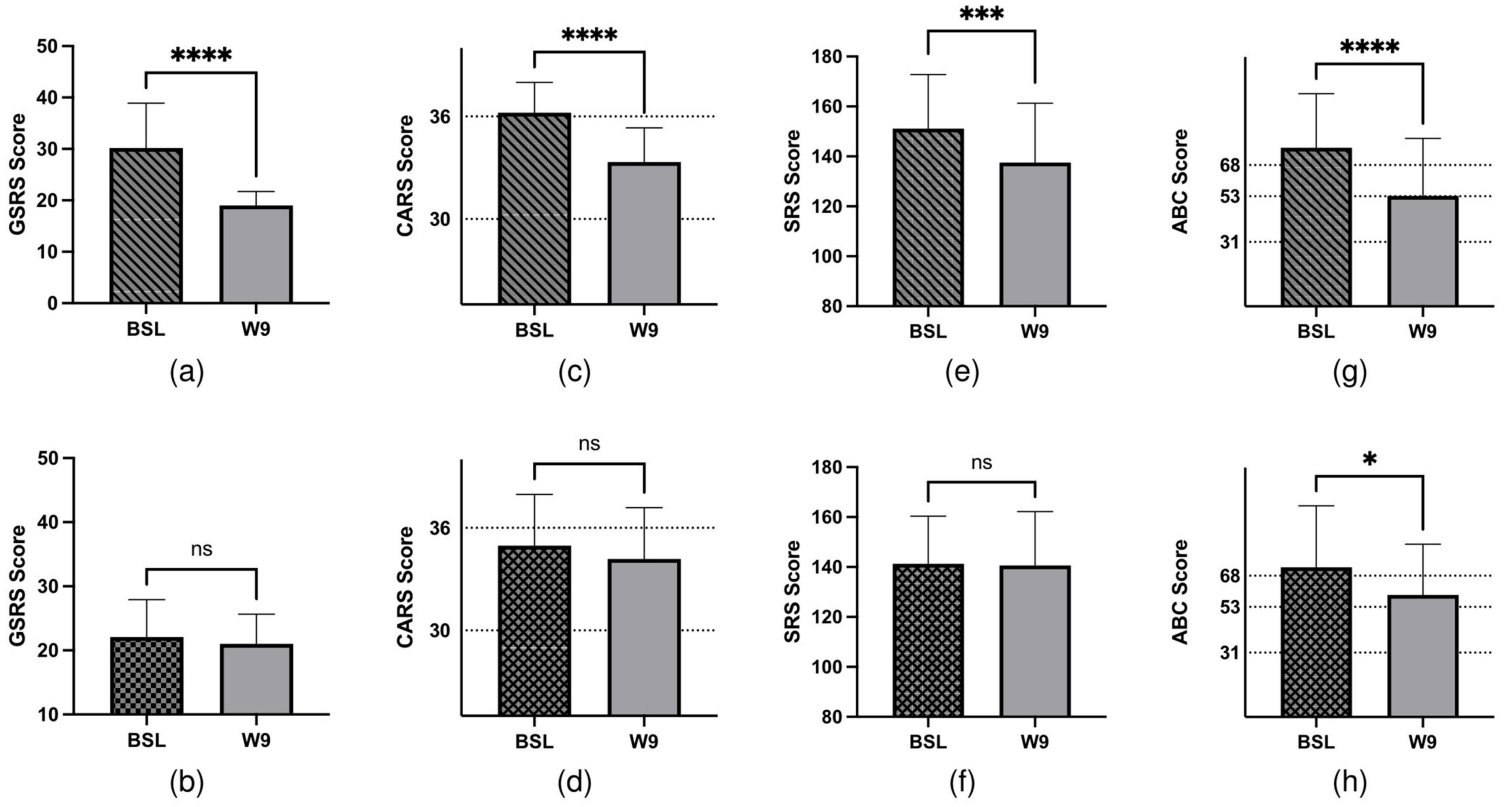
GI- and ASD-related symptoms of 41 children with ASD. Children were treated with FMT/placebo for 9 weeks. **(a)** Changes in GSRS scores after FMT treatment (*p* < 0.0001). **(b)** Changes in GSRS scores in the placebo groups (*p* > 0.05). **(c)** CARS assessment at baseline and post-treatment in the FMT groups (*p* < 0.0001). **(d)** CARS assessment at baseline and post-treatment in the placebo groups (*p* > 0.05). **(e)** Total SRS score at baseline and post-treatment in the FMT groups (*p* = 0.0002). **(f)** Total SRS score at baseline and post-treatment in the placebo groups (*p* > 0.05). **(g)** Total ABC score at baseline and post-treatment in the FMT groups (*p* < 0.0001). **(h)** Total ABC score at baseline and post-treatment in the placebo groups (*p* = 0.0342).

Significant improvements were observed in both GI and ASD symptoms following FMT. In the FMT group, GI symptoms, specifically diarrhea and constipation, showed notable improvement. The average GSRS scores decreased significantly from 30.17 (before FMT) to 19 (after FMT) (*p* < 0.0001), whereas the improvement in the placebo group was not significant (*p* = 0.19; Figures 1a,b). Similarly, the CARS average scores in the FMT group decreased from 36.22 to 33.33 (*p* < 0.0001), while the change in the placebo group was not significant (*p* = 0.053; Figures 1c,d). The SRS score showed a reduction from 151.17 to 137.5 in the FMT group (p < 0.0001), with no significant change observed in the placebo group (*p* = 0.782; Figures 1e,f). In addition, the ABC scores demonstrated a significant reduction in both groups, but the FMT group experienced a more pronounced decrease in component scores (*p* < 0.01; Figures 1g,h). To assess the safety of FMT, we also analyzed blood cell counts and liver and kidney function. The results indicated no abnormal indicators before or after the FMT intervention (Supplementary Table 1). However, after 9 weeks of FMT, alanine aminotransferase showed a significant increase, although both pre- and post-treatment levels remained within the normal reference range. This suggests that liver function should be closely monitored during the FMT process. In summary, FMT significantly improved both GI and autistic symptoms in children with ASD, demonstrating its potential as a beneficial treatment approach.

### 3.3 Effect of FMT on excretions of urine metabolites

**Figure 2:**
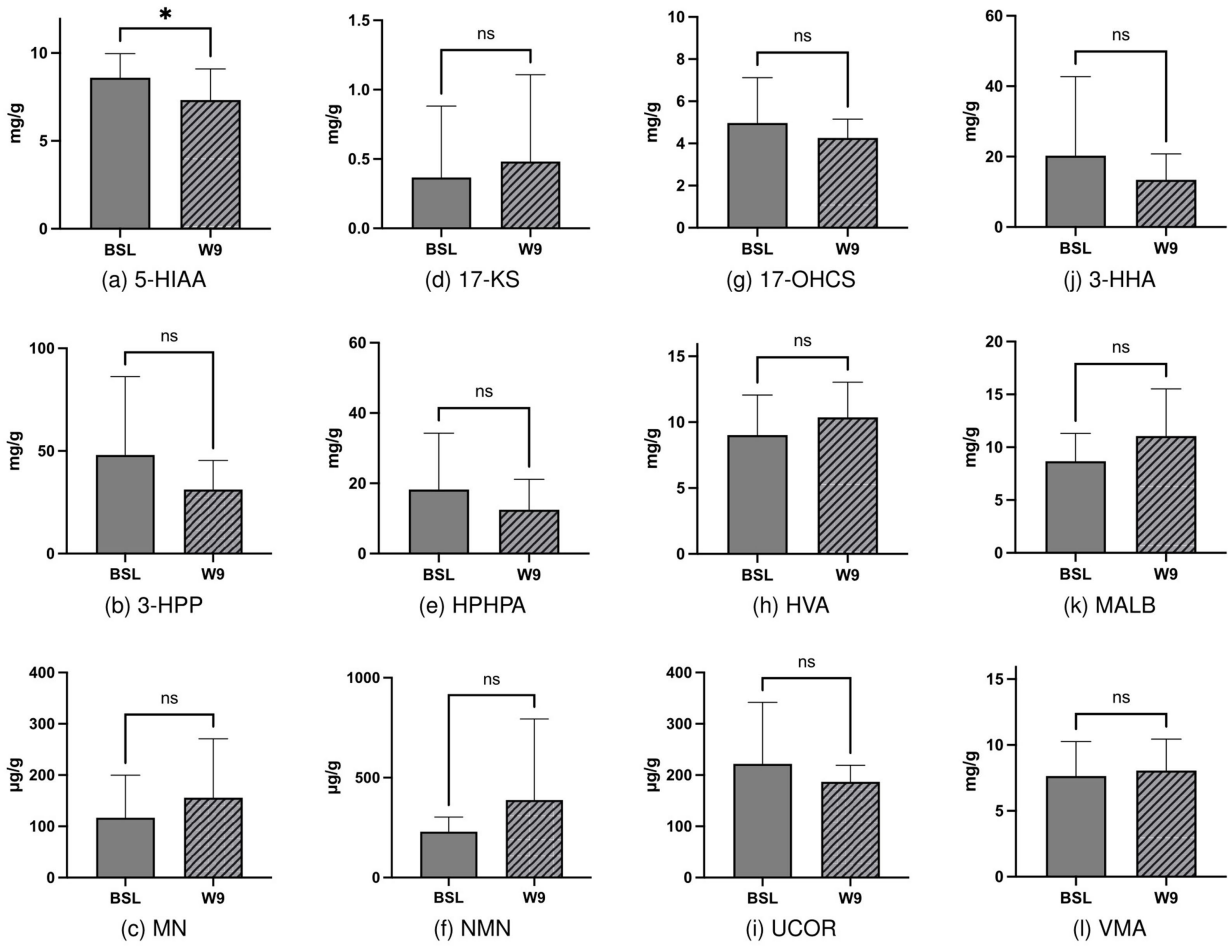
Changes in urinary metabolites before and after FMT intervention, with statistically significant differences observed in 5-HIAA before and after intervention (2a, *p* = 0.022).

Previous studies have reported the improvement of ASD and GI behaviors and changes in plasma metabolites after FMT. Therefore, we assessed the effects of FMT on urine metabolites. Evaluation of urine metabolites both at baseline and after the completion of FMT treatment revealed that among 12 urine metabolites, only 5-HIAA exhibited a significant decrease from 8.6 to 7.32 mg/L (*p* = 0.022; Figure 2). This result suggests that FMT may have a beneficial effect on 5-HT metabolism in individuals with ASD.

### 3.4 Correlation analysis of urine metabolites and clinical scales

**Figure 3:**
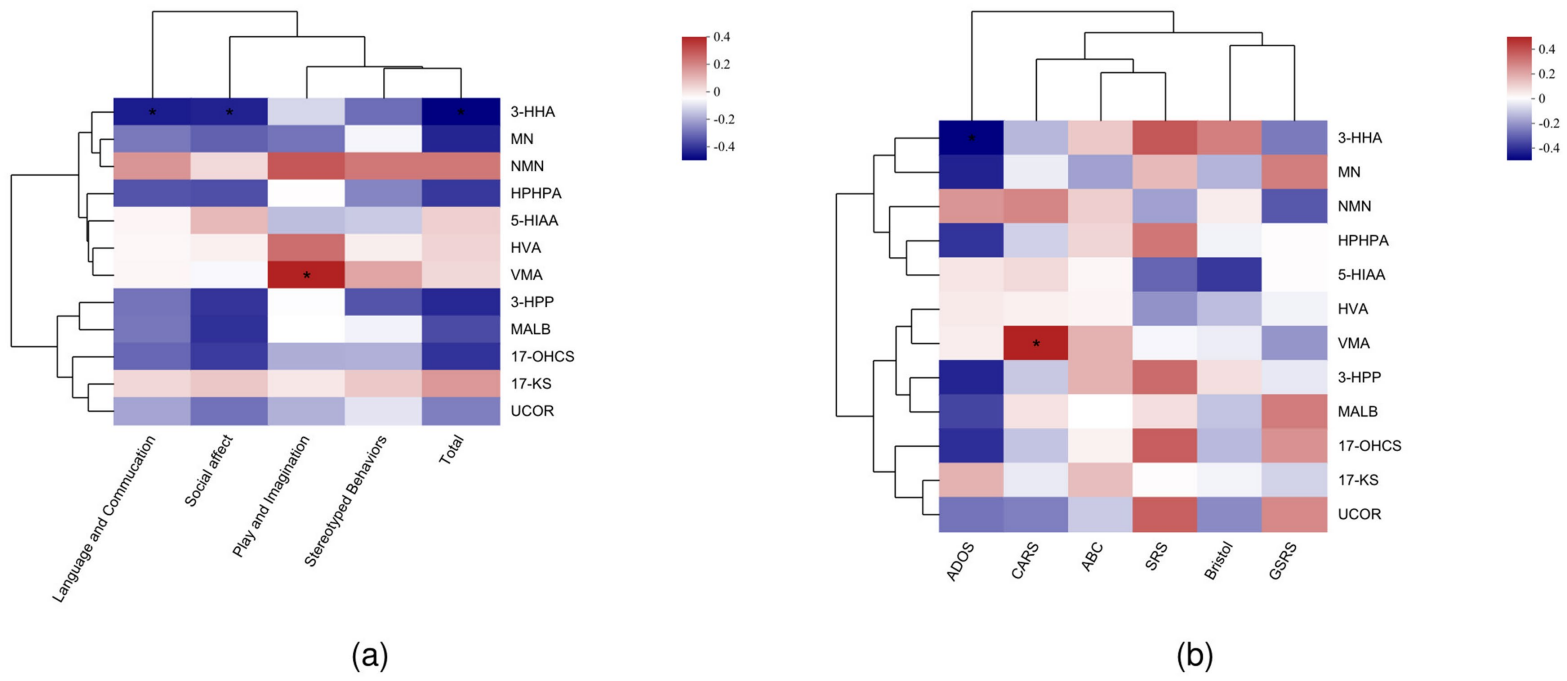
**(a)** Spearman’s correlation analysis of ADOS subgroups and urinary metabolites. **(b)** Spearman’s correlation analysis of clinical assessment scales and urinary metabolites.

Next, to explore the relationships between urinary metabolites and clinical symptoms, we performed Spearman’s correlation analysis. This analysis examined the associations between various domains of ASD symptoms and the concentrations of urine metabolites. Our findings showed that language and communication difficulties were significantly negatively correlated with 3-HHA (*p* < 0.05). Play and imagination skills showed a positive correlation with VMA (*p* < 0.05). In addition, social effect was negatively correlated with 3-HHA, and the total ASD score was also negatively correlated with 3-HHA (*p* < 0.05; Figure 3a). Furthermore, we analyzed the correlation between urinary metabolites and specific clinical scales such as CARS, ABC, and SRS. Our analysis revealed that the CARS scores were positively correlated with VMA (*p* < 0.05; Figure 3b).

## 4 Discussion

The effects of gut microbiota and metabolic disorders on ASD development have been demonstrated in several studies (14, 16). However, no standardized FMT intervention protocol for ASD exists. Clinically, altered urinary molecules such as 3-HHA, HPHPA, and 5-HIAA are commonly observed in children with ASD. In this study, we performed a randomized double-blind controlled trial to assess the clinical effects of FMT and explore the variations in urine metabolites before and after FMT interventions in children with ASD. Our findings demonstrated significant improvement in the GI and ASD-related symptoms with a significant decrease in the levels of 5-HIAA—a key metabolite of 5-HT metabolism, primarily excreted in urine. Moreover, the notable change in ABC scores observed within the placebo group can be attributed to the inherent subjectivity of the ABC questionnaire, which is based on parental reporting. This subjective evaluation is susceptible to various influencing factors, including parental perceptions and expectations. Conversely, the CARS employs an interview-based approach, where trained professionals conduct objective assessments. This methodology renders CARS a more reliable and consistent tool for symptom evaluation.

5-HT plays a pivotal role in both psychological and physiological regulation, contributing to the pathophysiology of conditions such as major depressive disorder, Alzheimer’s disease, schizophrenia, and ASD (20–22). In the context of tryptophan metabolism, most tryptophan is converted into kynurenine in the liver, with only a small fraction hydroxylated to 5-hydroxytryptophan (5-HTP), which is then decarboxylated to 5-HT in the brain. Some of this 5-HT is converted into melatonin, which regulates sleep, while another portion is metabolized into 5-HIAA and excreted via urine (23). Elevated 5-HIAA levels are indicative of dysregulated 5-HT metabolism in children with ASD. In this study, the observed reduction in 5-HIAA levels post-FMT highlights the possible role of the gut microbiota in regulating 5-HT metabolism. Prior studies have reported a negative correlation between 5-HT, 5-HIAA, and certain gut bacteria, such as Proteobacteria, suggesting an important interplay between 5-HT metabolism and the gut–brain axis (24).

In animal models, FMT has been shown to ameliorate autism-like behaviors induced by valproic acid by modulating gene expression in serotonergic synapse pathways in the colon. These pathways are enriched in metabolites such as oxidized L-proline, L-asparagine, and thromboxane B2 (25). In addition, germ-free animal models demonstrate increased levels of tryptophan and decreased 5-HT, reinforcing the notion that gut microbiota directly influences tryptophan and 5-HT metabolism (26, 27). Gut microbiota is also known to regulate the expression of tryptophan hydroxylase (TPH1/TPH2), which modulates 5-HT biosynthesis through short-chain fatty acids (28). Our findings further support the hypothesis of a complex relationship between serotonin metabolism and gut microbiota, which may be modulated by FMT.

Correlation analysis also identified that 3-HHA and VMA are more closely associated with the clinical manifestations of autism than 5-HIAA. 3-HHA is a byproduct of phenylalanine metabolism, primarily influenced by gut bacteria such as *Clostridium difficile*. An overgrowth of *C. difficile* leads to the conversion of phenylalanine to meta-tyrosine, which is subsequently transformed into 3-hydroxyphenylalanine through deamination and further oxidized into 3-HHA (29). This harmful metabolite has been implicated in the pathogenesis of ASD. VMA, the final metabolite of the catecholamine pathway, is similarly affected by toxic microbial byproducts, including HPHPA and 3-HHA, derived from *C. difficile* (30). Abnormal levels of these metabolites and their correlations with ASD symptoms reflect the significant role gut microbiota plays in the disorder.

Despite the promising findings of this study, several limitations should be noted. First, the sample size was relatively small, which may affect the generalizability of the results. Larger, multicenter trials are necessary to confirm the observed improvements in gastrointestinal and ASD-related symptoms following FMT. Second, while urinary metabolite analysis provides valuable insights into systemic changes, it may not fully capture the complexities of central serotonin metabolism. Given that 5-HIAA levels in urine may not directly reflect serotonin concentrations in the brain, additional measures, such as cerebrospinal fluid sampling, would be necessary to better understand central nervous system serotonin dynamics in children with ASD. Another limitation is the lack of a long-term follow-up to assess the sustainability of the therapeutic effects of FMT on both gut microbiota and ASD symptoms. It remains unclear whether the observed benefits persist over time or whether repeated treatments are required. Furthermore, while correlations between certain metabolites and ASD symptoms were identified, the study did not establish direct causality. Additional studies, including animal models and more comprehensive microbiome analyses, are needed to explore the mechanisms linking gut microbiota and metabolic dysregulation to ASD pathogenesis. Finally, the focus of the study on urinary metabolites may have missed other relevant biochemical pathways or metabolites that contribute to ASD symptomatology, which could have provided a more holistic understanding of the disorder.

This randomized, double-blind controlled trial suggests that FMT may be a promising therapeutic approach for alleviating gastrointestinal and ASD-related symptoms in children. Our findings indicate that FMT can significantly reduce urinary 5-HIAA levels, suggesting a potential role of the gut microbiota in regulating serotonin metabolism, which is often dysregulated in individuals with ASD. The study also highlights the importance of metabolites such as 3-HHA and VMA, which were found to correlate more strongly with ASD symptoms, further implicating gut microbiota in the metabolic dysfunction observed in the disorder. Nevertheless, larger-scale studies and mechanistic research are required to fully elucidate the relationship between gut microbiota, metabolic pathways, and ASD. Future research should explore central nervous system serotonin metabolism and consider longer-term follow-ups to assess the durability of the effects of FMT. In conclusion, this study provides preliminary evidence that FMT could be a valuable addition to the therapeutic options for children with ASD, especially in cases where gastrointestinal symptoms are prevalent, but further research is needed to confirm these outcomes and refine treatment protocols.

## Data Availability

The original contributions presented in the study are included in the article/Supplementary material, further inquiries can be directed to the corresponding author.

## Supplementary material

SUPPLEMENTARY TABLE 1 Routine blood tests and assessments of liver and kidney function.
