# Supplementary material for: FMT intervention decreases urine 5-HIAA levels: a randomized double-blind controlled study
Source: Front Med (Lausanne). 2024 Oct 18;11:1411089. doi: 10.3389/fmed.2024.1411089 (PMC11529335; doi:10.3389/fmed.2024.1411089)
Supplement: SUPPLEMENTARY TABLE 1 — Routine blood tests and assessments of liver and kidney function. [file Table_1.DOCX]

| Clinical indicators | FMT BSL | FMT W9 | P value |
| --- | --- | --- | --- |
| Hemoglobin  White blood cells  Red blood cells  Platelets  Aspartate aminotransferase  Alanine aminotransferase  Total bilirubin  Direct bilirubin  Albumin  Globulin  Blood urea nitrogen  Creatinine | 131.07(126.00,137.50)  8.20(6.19,9.20)  4.72(4.45,4.97)  349.69（282.00，417.00）  18.46（11.75，23.50）  15.92（10.75，20.25）  6.51（4.95，8.00）  2.41（1.90,3.03）  47.63（45.75，50.15）  22.18（20.58，23.70）  4.07（2.9，5.05）  37.88（34.75，41.25） | 128.72(124.50,135.50)  7.80(6.31,9.02)  4.65(4.52,4.85)  353.07(301.00,394.00)  17.14(8.75,21.75)  24.22(12.50,26.25)  6.50(4.58,9.05)  2.47(2.00,3.10)  46.97(45.00,49.00)  22.64(20.10,24.08)  4.08(3.15,4.88)  37.13（31.83,42.00） | 0.301  0.717  0.369  0.454  0.319  **0.011**  0.735  0.742  0.918  0.737  0.528  0.539 |

| Clinical indicators | Placebo BSL | Placebo W9 | P value |
| --- | --- | --- | --- |
| Hemoglobin  White blood cells  Red blood cells  Platelets  Aspartate aminotransferase  Alanine aminotransferase  Total bilirubin  Direct bilirubin  Albumin  Globulin  Blood urea nitrogen  Creatinine | 133.83(127.50,142.00)  8.59(7.83,9.22)  4.78(4.47,4.97)  384.08（316.75，420.00）  14.50（10.25，15.75）  18.25（10.50，28.75）  7.44（5.08，9.30）  2.76（2.03,3.35）  47.53（45.23，51.20）  22.90（22.10，23.80）  4.44（3.85，5.40）  38.83（33.50，45.00） | 129.17(124.00,135.00)  8.08(6.91,9.21)  4.65(4.51,4.92)  372.50(309.50,425.50)  16.56(8.5,16.25)  24.31(13.25,29.50)  8.23(5.43,9.58)  3.08(2.33,3.50)  45.61(43.18,46.83)  22.77(20.38,24.78)  4.20(2.90,5.25)  36.28（31.08,40.75） | **0.042**  0.386  0.131  0.448  0.754  0.154  0.499  0.637  0.149  0.812  0.524  0.063 |
